# Telomere Length Dynamics and the Evolution of Cancer Genome Architecture

**DOI:** 10.3390/ijms19020482

**Published:** 2018-02-06

**Authors:** Kez Cleal, Kevin Norris, Duncan Baird

**Affiliations:** Division of Cancer and Genetics, School of Medicine, UHW Main Building, Cardiff CF14 4XN, UK; clealk@cardiff.ac.uk (K.C.); norriskt@cardiff.ac.uk (K.N.)

**Keywords:** telomeres, fusions, telomerase, ALT, genome instability, cancer

## Abstract

Telomeres are progressively eroded during repeated rounds of cell division due to the end replication problem but also undergo additional more substantial stochastic shortening events. In most cases, shortened telomeres induce a cell-cycle arrest or trigger apoptosis, although for those cells that bypass such signals during tumour progression, a critical length threshold is reached at which telomere dysfunction may ensue. Dysfunction of the telomere nucleoprotein complex can expose free chromosome ends to the DNA double-strand break (DSB) repair machinery, leading to telomere fusion with both telomeric and non-telomeric loci. The consequences of telomere fusions in promoting genome instability have long been appreciated through the breakage–fusion–bridge (BFB) cycle mechanism, although recent studies using high-throughput sequencing technologies have uncovered evidence of involvement in a wider spectrum of genomic rearrangements including chromothripsis. A critical step in cancer progression is the transition of a clone to immortality, through the stabilisation of the telomere repeat array. This can be achieved via the reactivation of telomerase, or the induction of the alternative lengthening of telomeres (ALT) pathway. Whilst telomere dysfunction may promote genome instability and tumour progression, by limiting the replicative potential of a cell and enforcing senescence, telomere shortening can act as a tumour suppressor mechanism. However, the burden of senescent cells has also been implicated as a driver of ageing and age-related pathology, and in the promotion of cancer through inflammatory signalling. Considering the critical role of telomere length in governing cancer biology, we review questions related to the prognostic value of studying the dynamics of telomere shortening and fusion, and discuss mechanisms and consequences of telomere-induced genome rearrangements.

## 1. Introduction

**Figure 1:**
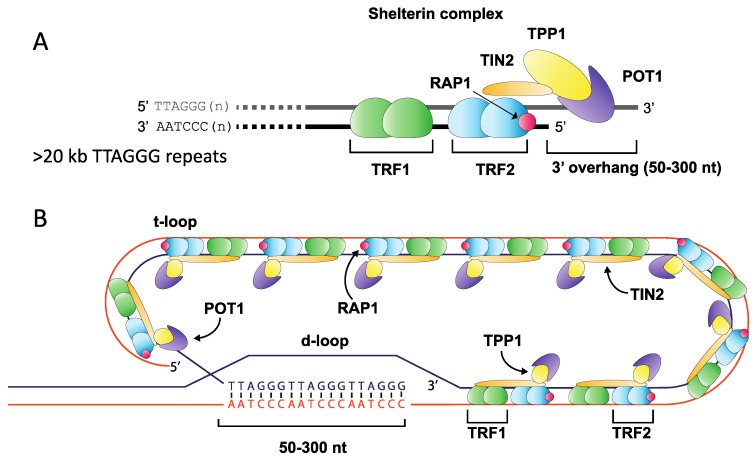
Overview of telomere structure. The terminal ends of mammalian chromosomes consist of an array of (TTAGGG)_n_ repeats ending with a 3′ overhang of between 50 and 300 nt in length (**A**). This array is bound by many protein components including members of the shelterin complex, which anchors to the repeat array through Telomere Repeat binding Factors 1 & 2 (TRF1 and TRF2), binding repeats as a homodimer, and forming a complex with TIN2 (TRF1-interacting factor), RAP1 (Repressor Activator Protein 1), TPP1 and POT1 (Protection of Telomere 1) (**A**). The repeat array folds into a higher-order t-loop structure where the 3′ overhang displaces a portion of the forward strand to create a d-loop, thereby sequestering the free chromosome end from the DNA repair machinery (**B**).

Telomeres play a fundamental role in maintaining eukaryotic genome integrity by ensuring that the natural ends of chromosomes are not mistakenly recognised as double-stranded DNA breaks (DSB) and processed by DNA repair pathways [1]. The highly-conserved telomere repeat tract found at the ends of human chromosomes consists of tandem repeats (TTAGGG)_n_, terminating with a 3′ overhang that is created via nucleolytic degradation [2,3,4]. This 3′ overhang undergoes strand invasion into the repeat array, assembling into a higher-order chromatin structure that consists of a t-loop and telomere-associated protein components including the shelterin complex (TRF1 and TRF2, POT1, TIN2, RAP1 and TPP1) [5,6]. This specialised structure represses DNA damage signalling, preventing activation of the ATM and ATR kinases, which may otherwise induce cell cycle arrest and promote DNA repair (Figure 1) [7,8,9,10].

Due to the inability of the DNA replication machinery to fully replicate linear DNA (the end replication problem), telomeres progressively shorten with each cell division [11,12]. Telomeres may be elongated by the telomerase complex, which consists of the catalytic telomerase reverse transcriptase (TERT) subunit and telomerase RNA component (TERC), the RNA template from which TERT can actively add telomere repeats to the chromosome terminus [13,14,15,16]. Whilst TERC is expressed widely, TERT expression is downregulated in somatic cells, leading to progressive telomere shortening [13,14,15].

In most cells, very short telomeres lead to senescence and cell cycle arrest [17]. In humans, this is thought to represent a fundamental tumour suppressor mechanism that limits the proliferative capacity of a cell, whilst also preventing the genome instability that may arise from telomere dysfunction [5,11]. This mechanism appears to be more common in long-lived and larger organisms, with mice, for example, possessing considerably longer telomeres than humans, and telomerase activity appearing to be less stringently regulated in mouse somatic cells [18,19,20,21]. Curiously, in humans, both excessively long and short telomeres have been associated with increased cancer rates, suggesting that there is a sweet spot for mean telomere length in preventing oncogenesis [22]. The mechanism by which longer telomere length leads to increased cancer risk remains unclear, although it is plausible that setting the telomere length too long permits an increased replicative lifespan for a cell that extends the window over which transformation may occur. On the other hand, excessively short telomeres may drive too many cells into senescence, increasing the odds of bypassing this barrier and leading to telomere dysfunction. Additionally, senescent cells have further deleterious effects on the tissue microenvironment through the acquisition of a senescence-associated secretory phenotype (SASP) that can promote tumour progression through proinflammatory signalling [23,24,25].

In human somatic cells in which DNA damage checkpoints are compromised, continued cell division and consequent telomere loss past the point at which senescence normally occurs lead to a phase known as “crisis” that is characterised by telomere uncapping, subsequent fusion events, and widespread genome instability [26,27]. The dynamic loss and gain of whole chromosomes or segments of DNA is an early occurrence in cancer progression and is thought to be crucial for generating diversity in premalignant clones and driving oncogenic transformation through natural selection [28]. Possession of a genetic signature that confers a fitness advantage over a competitor leads to clonal expansion, and reiteration of this process can lead to a clonal cell population with a radically altered genetic profile, exhibiting oncogene overexpression and disrupted regulation of tumour suppression pathways [28]. Crucially, an emerging tumour-initiating clone requires the activation of a telomere length maintenance pathway in order to escape crisis, which is only achieved through either reactivation of telomerase found in around 85–95% of cancers, or through elongation of telomeres via the ALT pathway [29,30,31,32,33,34].

This review is focused on telomere length dynamics in the context of cancer progression. We discuss how telomere dysfunction can lead to the evolution of an immortalised clonal population of cells with a radically altered karyotype and disrupted oncogene or tumour suppressor pathways. Additionally, the prognostic potential of telomere length or dysfunction is considered, and how this can be utilised to improve and personalise patient treatment course.

## 2. Telomere Length Dynamics

Telomere length is a species-specific trait that shows considerable variability between individuals as well as between tissues and across different chromosome arms [19,35,36]. In humans, telomere length is to a large extent genetically determined and shows a high degree of heritability with correlations between mother-offspring telomere length and a positive association with paternal age [37,38]. Though telomeres typically range in size from 5 KB to 12 KB in early adulthood, cross-sectional studies indicate that differences in their length among somatic tissues are largely established in the first two decades of life, which is thought to echo the underlying developmental program of the tissue [39].

The length of telomeres is carefully regulated, with recent studies pointing to a telomere trimming mechanism involving telomeric zinc finger-associated protein (TZAP), which sets an upper limit on telomere length [40,41]. At shorter telomeres TZAP is outcompeted for binding by TRF1 and TRF2, although diluted binding of these shelterin components at longer telomeres allows for binding of TZAP and promotion of telomere trimming [42]. Each chromosome may also display its own characteristic length profile, and distinctions are often observed between different alleles within the same cell, giving rise to a bimodal population of telomeres at a given chromosome end [36,43]. Inter-allelic differences have been observed as large as 6.5 KB in normal fibroblasts, underlining the potential heterogeneity of telomere lengths within a single cell [36].

In somatic stem cells and some immune cells, telomerase activity is detectable at low levels; however, this activity is clearly insufficient to maintain telomere length, as progressive telomere losses are observed during aging [14,15,44]. In most cells, telomerase insufficiency or silencing leads to progressive telomere erosion on the order of 50–100 bp per cell division, or (at the tissue level) around 24 ± 7 bp per year in leukocyte, muscle, skin and fat cells [29,39,45]. By adulthood, rates of telomere erosion show strong intra-individual synchrony irrespective of the proliferative demands of the tissue, which implies that attrition rates in stem cell populations are relatively consistent among tissues and proliferative demand is met through expansion of progenitor cells rather than increased stem cell division [39].

In vitro, subsets of telomeres have been observed with lengths far below the sample mean, consistent with a rapid deletion of the repeat array. These stochastic events were observed with a frequency of around 4% at the XpYp telomere resulting in telomeres with a length <2.32 standard deviations below the sample mean with potentially dysfunctional characteristics [36,43]. Interestingly, cells with grossly shortened telomeres did not accumulate over time, suggesting those cells had exited the cell cycle, repaired their telomeres or telomere fusions had occurred, preventing further detection [36,43]. However, it is unclear to what extent rapid telomere shortening occurs in vivo and whether it plays a role in driving genomic instability and pathology.

## 3. Telomere Crisis and Escape

In cell culture, telomere crisis can be induced by ablation of shelterin components such as TRF2, or by overexpression of a dominant-negative telomerase construct (DN-hTERT), followed by prolonged cell culture to induce replicative telomere erosion [46,47]. DN-hTERT expression is thought to more closely resemble in vivo conditions during tumour development, although a drawback of the system is that escape may be achieved by a reduction in overexpression of the vector, in the absence of more biologically relevant events such as a gain in copy number of wild-type telomerase, or the release of epigenetic repression.

Initially, continual passage of cells that lack functional telomerase leads to progressive erosion of telomere length and a concomitant increase in the variance of the distribution consistent with random cell division and exponential growth [26]. However, as telomeres shorten past a critical threshold, the growth kinetics begin to change, marking the inception of crisis, a state characterised by high numbers of senescent cells, morphological changes, reduced doubling rate, high levels of cell death through apoptosis or necrosis and widespread genome instability [48,49]. During this period, telomere fusions may also be detected, involving telomere–telomere events or joins with other genomic loci [26,50,51]. As crisis progresses, growth may cease entirely and remain that way indefinitely or until all cells have perished [26]. Escape from crisis is then only achieved through reactivation of telomerase, or through the ALT pathway [29,30,31,32,33].

Recurrent point mutations in the promoter of TERT have been identified in a range of cancers, with an overall frequency of around 19%, although some cancers show a higher incidence [52,53,54,55,56,57,58]. Multiple transcription factor families have binding sites at the TERT promoter and several studies suggest that the chromatin environment plays a role in telomerase reactivation [59,60,61]. A G-quadruplex motif was recently found to play a role in maintaining repressive chromatin at the TERT promoter through the action of metastatic suppressor non-metastatic 2 (NME2) protein, which appeared to mediate recruitment of silencing factors [60]. Large-scale structural variation, including balanced rearrangements, translocations and focal, high level amplifications, has also been documented at the TERT locus in a subgroup of high-risk neuroblastoma patients, suggesting that remodelling of the genomic context may lead to release of transcriptional silencing [61].

Telomere stabilisation by the ALT pathway relies on homologous recombination to elongate telomeres through a mechanism that is thought to resemble repair by the break-induced replication (BIR) pathway [30,62,63,64]. Somatic mutations in the alpha-thalassemia X-linked syndrome protein (ATRX), histone variant H3.3, and the death associated protein (DAXX) have been identified in ALT positive cancers [65,66]. The consequences of elongation of telomeres by ALT differ according to the actions of telomerase in several respects. ALT cells tend to display a highly heterogeneous telomere length profile, with both short and very long telomeres possible within a single cell [67]. Fluctuating telomere lengths and frequent exchange between sister chromatids have also been observed [68,69]. Additionally, ALT cells exhibit APBs (ALT-associated promyleocytic leukaemia bodies) and C-circles—extensive extra-chromosomal telomere repeats that consist of partially single stranded circles of C-strand DNA derived from telomeres [70,71]. Interestingly, ALT-elongated telomeres continue to experience replicative erosion and so periodic rounds of recombination may be required to maintain immortality, or ALT tumours may recourse to reactivation of telomerase.

The interplay of these two pathways is also beginning to be explored, with recent studies suggesting that telomerase may play an important role in ALT inhibition, with the implication that antitelomerase therapies may inadvertently promote ALT induction [72,73]. 

## 4. Telomere Fusions

Telomeres that lack sufficient protection from the DSB repair machinery may undergo fusions with other telomeres in a head-to-head orientation or with other non-telomeric genomic loci following an additional genomic break. By studying the structure of telomere fusions at the nucleotide level, recent studies indicate that the classical and alternative non-homologous end joining pathways (C-NHEJ and A-NHEJ, respectively) play a central role in mediating these joins [47,51,74,75].

The C-NHEJ pathway typically mediates blunt-end ligation, involving the high affinity binding of Ku proteins to either side of the break, which promotes interaction with the X-ray Cross Complementing: DNA ligase IV complex (XRCC4:LIG4) to catalyse the join [74,76,77,78]. C-NHEJ is a high-flux pathway that is responsible for repairing the bulk of cellular DSBs, with fusions characterised by low levels of break site processing and random levels of microhomology at the join. Although C-NHEJ can join a broad spectrum of breaks, the structure of some DNA ends presents a problem for this pathway. In particular, breaks with single-strand overhangs bind only weakly to the Ku recognition factors, inhibiting repair by the C-NHEJ pathway and thereby favouring A-NHEJ repair [79]. The A-NHEJ pathway exhibits a trend towards increased levels of microhomology and end resection at break sites [74,80,81], and employs DNA polymerase θ, a mutagenic polymerase that can generate templated insertions at break sites [82]. End joining by the A-NHEJ pathways is performed primarily by DNA-Ligase III (LIG3), although DNA-Ligase I (LIG1) may also be utilised in some cases [51,83,84].

We have investigated the consequences of deficiencies in A- and C-NHEJ in mediating telomere fusions by the knockout of ligases LIG3 and LIG4 (LIG3^−/−^, LIG4^−/−^). In the context of DN-hTERT overexpression to drive cells into crisis, wild-type and LIG4^−/−^ cells displayed telomere fusions during crisis and readily escaped, though LIG4^−/−^ cells displayed a reduction in the number of inter-chromosomal events between the 17p and XpYp telomere [26,51]. Strikingly, LIG3^−/−^ cells also displayed telomere fusions but were uniformly unable to escape crisis, with the entire culture perishing within two to three months [26]. Interestingly, LIG3^−/−^ cells showed a significantly higher proportion of inter-chromosomal events (17p to XpYp fusions) relative to wild-type and LIG4^−/−^ cells.

These studies suggested that telomere fusions effected by the C-NHEJ pathway may therefore be more deleterious to the cell, presumably due to increased numbers of inter-chromosomal events that constitute a more severe disruption to the genetic and epigenetic landscape. Conversely, telomere fusion mediated via the A-NHEJ pathway may provide a selective advantage during crisis by favouring intra- or sister chromatid rearrangements that may result in localised amplification or deletion events. These observations raise the possibility of selectively targeting the A-NHEJ pathway during cancer progression to preclude the escape from crisis.

Although less studied, homologous recombination (HR) pathways may also play a role in telomere fusions. In cells lacking the shelterin components TRF2 and RAP1, the homologous recombination factors PARP1 and SLX4 promoted resection of telomeres resulting in widespread telomere-free fusions involving nearly 50% of chromosome ends [85]. We hypothesise that additional pathways such as the BIR pathway may also mediate telomere fusions at short dysfunctional telomeres, possibly driven by template switching events through the microhomology mediated break induced replication (MMBIR) mechanism [86], although this remains to be investigated directly.

Whilst the end joining pathways that mediate telomere fusions are beginning to be elucidated, characterising the end-processing that occurs prior to repair has received less attention. From sequencing the break sites of end-to-end telomere fusions sister chromatids appeared to be differentially processed with one sister chromatid often undergoing a deletion event before joining [50,51]. The longer chromatid often showed telomeric repeats but was joined with the shorter chromatid, several kb into the sub-telomere region, with greater distances obscured by limitations of the assay [26,50,51,87]. These observations indicate that one of the sister chromatids was subjected to rapid resection prior to joining, although alternative mechanisms and means of repair cannot be ruled out.

Telomere fusions have been shown to involve loci throughout the genome although the factors that govern where a fusion may occur remain poorly understood [51]. There is an expectation that chromatin accessibility, and potentially transcriptional activity at a specific locus, will increase the probability of a telomere fusion due to the increased likelihood of provision of a DSB fusion partner for the dysfunctional telomere at these loci. Additionally, the organisation of chromatin within the nucleus may play a role by favouring repair between local DSB ends. Other genomic features such as non-B-form DNA secondary structures, fragile sites or interstitial telomere repeats may also potentially enhance the occurrence of telomere fusions, and the stage of the cell cycle may further constrain the types of events that may arise [87,88].

## 5. Telomere Fusions as Drivers of Genome Instability

Complex genome rearrangements can occur early during the progression to malignancy, preceding the invasive and metastatic stage of the disease, and are evident by the time telomerase expression is detected [89,90,91]. Telomere driven genome instability is considered to be intimately linked to oncogenesis by fostering clonal diversity and evolution of the genome through copy number gains or losses, genome reorganisation and chromatin remodelling effects [92]. Investigating the consequences of telomere fusions on genome architecture therefore provides insight into the mechanisms that govern cancer progression.

Dysfunctional telomeres can be fused with other loci in various configurations giving rise to sister chromatid, intra- or inter-chromosomal events. As replicative erosion occurs in parallel at all 92 telomeres of the cell, telomere dysfunction and telomere induced DNA damage signalling may occur rarely in isolation and instead may arise simultaneously at multiple ends. Additionally, the inception of telomere dysfunction may be most likely immediately following the telomere losses that occurs during DNA replication, when a dysfunctional telomere will be spatially prearranged with a sister chromatid with similar, potentially dysfunctional characteristics. Fusions between sister chromatids in end-to-end joining events are thus predicted to be more prevalent than inter-chromosomal end-to-end events, although limitations with current analysis techniques make this assumption difficult to prove [50,87,93]. In the TRF2 knockdown model to induce telomere dysfunction, fusions were found to predominate between acrocentric chromosomes (chromosomes possessing a centromere located near the end of the chromosome), creating stable dicentric chromosomes (chromosomes with two centromeres) that persisted for months in cell culture, although it is unclear if this finding extends to models of telomere dysfunction driven by replicative erosion [94].

**Figure 2:**
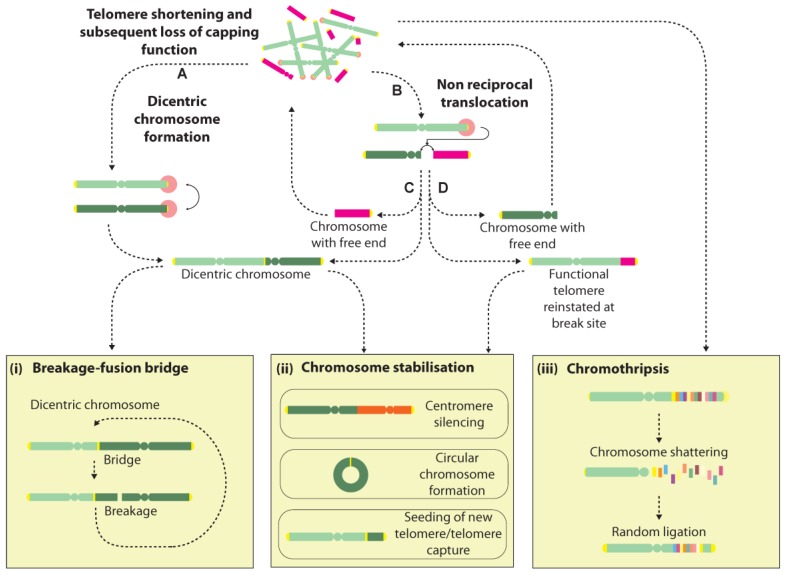
Cascades of genome rearrangement induced by free chromosome ends. Telomeres that lose their capping function can trigger DNA damage signalling and subsequent processing by DSB repair pathways, instigating genome instability via several mechanisms. Telomere-telomere (**A**) fusions create dicentric chromosomes that may undergo rupture during cell division, giving rise to a succession of events in a BFB cycle (**i**). Alternatively, dicentric chromosomes may be stabilised by centromere silencing (**ii**). Dysfunctional telomeres may also undergo joining with internal genomic loci giving rise to non-reciprocal translocations (**B**). Depending on the nature of the join, several outcomes are possible with examples of events given in C&D. One outcome is the formation of a dicentric chromosome plus an acentric chromosome (**C**), which may elicit further BFB events or instability by reintegration of the acentric chromosome at another genomic site. Alternatively, a functional telomere may be transferred to the initiating chromosome, at the cost of creating another free end at the participating chromosome (**D**). Chromosomes with free ends may be stabilised by circularisation, or through the seeding of a new telomere (**ii**). Recent reports suggest that telomere dysfunction may also trigger “all-at-once” events such as chromothripsis, a process thought to involve the shattering and random re-ligation of chromosomes that may either give rise to a stable genomic configuration, or may promote further instability due to the presence of a free chromosome end (**iii**).

Dicentric chromosome formation is one potential consequence of a telomere fusion event. They can be unstable structures that may be broken during cell division and initiate further rounds of fusion and breakage, leading to a cycle of events in what is known as the breakage-fusion-bridge (BFB) cycle [27,95] (Figure 2). Dicentric chromosomes can additionally arise from telomere fusions with non-telomeric genomic loci, though with the additional consequence of creating an acentric chromosome, which may induce further genomic rearrangements and instability [96]. Rupture of the conjoining sequence between the two centromeres at cell division can lead to several classes of rearrangements including deletions, duplications and translocations. Extended BFB cycling can generate extremely convoluted genome rearrangements and is often invoked to explain the amplified inverted repeats seen in solid tumours [97,98,99,100] (Figure 2).

The genomic context of breakage during a BFB cycle is poorly defined, and may depend on several factors relating to the local chromatin environment through fragility effects, or may be related to sequence context or the presence of DNA lesions and modifications [101,102]. In yeast, dicentric breakage occurs during cytokinesis with cleavage occurring preferentially at both the pericentromeric region and intriguingly at the initiating telomere fusion site [103]. Thus, yeast appears to have some capacity to recover its normal karyotype in the event of a telomere-telomere fusion, although it is unknown if this mechanism exists in humans.

Another possible consequence of a telomere fusion is the creation of a non-reciprocal translocation that results in the transfer of the distal portion of one chromosome arm to the dysfunctional telomere, or the partial copy of one chromosome by the break-induced replication pathway [104,105]. This transfer potentially reinstates a functional telomere at the initiating chromosome whilst the participating chromosome is left with a free end that may undergo repair with another locus, raising the possibility of a cascade of events that may only be terminated by the formation of a dicentric or circular chromosome, or the seeding of a new telomere at the free end, potentially via a recombination event (Figure 2). Thus, the progressive genomic instability fostered by BFB cycling over extended cell generations is thought to partly underlie the highly-rearranged genomes seen in cancer [27,106].

However, several groups have recently raised the prospect that telomere-induced genome instability may have an immediate and even more spectacular consequence on the integrity of the genome, by instigating a genome catastrophe—a whole-genome restructuring event that occurs in a single step [100,107,108,109] (Figure 2). Several classes of genome catastrophe have been proposed that ostensibly describe the differing genomic architectures and patterns identified, including chromothripsis, chromoplexy and chromoanasynthesis [107,108,109,110,111]. Chromothripsis is by far the most widely reported pattern, and is thought to arise from a chromosome shattering event that affects one or multiple chromosome arms [107]. Chromosome fragments are thought to undergo random ligation with concomitant loss of some fragments, giving rise to a shuffled pattern interspersed by regions showing loss of copy number or loss of heterozygosity [111]. Recently, the phenomenon of chromothripsis was reported in RPE1 cells following TRF2 knockdown to induce telomere dysfunction [112]. Tantalisingly, dicentric bridges were seen frequently in cells during crisis, and sequencing of post-crisis clones with abnormal karyotypes revealed a high incidence of chromothripsis, suggesting that chromothripsis may arise during dicentric bridge resolution during cell division (Figure 2).

## 6. Telomere Length as a Prognostic Marker in Cancer

The promotion of large scale genome rearrangements and instability by telomere dysfunction has been well documented [51,72,113,114,115,116]. Telomere shortening, which leads to these events, is an early event during neoplastic progression [116,117,118,119,120]. These findings have led to significant interest as to whether telomere length could be used as a prognostic marker to determine clinical outcome in cancer [117,121].

Chronic Lymphocytic Leukaemia (CLL) has provided an example of the use of telomere length measurements to define prognosis. CLL has a heterogeneous clinical course with overall survival ranging from a few months to many decades [122]. A multitude of clinical markers now exist for CLL including disease stage, patient age and performance status to numerous molecular markers such as immunoglobin gene mutational status, cytogenetics, CD38 expression and ZAP70 expression [123,124]. Although each marker used alone can give an indication of what happens to a subset of patients, none can give a definitive individual prognosis for a patient [125]. However, the use of high-resolution telomere length analysis identified telomere shortening and an increase in frequency of telomere fusion events during CLL progression [116]. Importantly, a subset of severely shortened telomeres, and associated telomere fusions, were also found in early-stage patient samples, indicating that these events precede disease progression [117]. Concomitant with telomere dysfunction in CLL patients with shorter telomeres, was the occurrence of large-scale genome rearrangements that were concentrated at telomeric regions [116]. Importantly, this was not observed in patients with longer telomeres. The telomere dynamics observed in CLL B cells was indistinguishable from cells undergoing crisis in culture following abrogation of the p53 pathway [116].

The telomere length below which telomere fusion was detected was established in CLL B-cells. This “fusogenic” range was then used to stratify patients into favourable and unfavourable prognosis categories, even in early stage patients [117]. Patients with telomeres above the fusogenic mean showed superior prognosis regardless of their IGHV mutation status or cytogenetic risk group. In keeping with these findings, telomere length was the dominant variable in multivariate analysis [117,121]. Telomere length has also been shown to be superior to established and recently discovered genomic biomarkers for predicting prolonged progression free survival following chemotherapy [121]. Taken together, these findings demonstrate telomere length as a powerful prognostic and predictive marker for CLL.

Interestingly, use of this fusogenic range prognostic tool also appears to successfully stratify patients into favourable and unfavourable disease progression categories in breast cancer [126]. When a similar approach was performed in a cohort of tumour samples isolated from patients with invasive ductal carcinoma of the breast, a subset of patients were identified with a mean telomere length less than the fusogenic threshold. This subset of patients displayed a poor clinical outcome with a median survival of less than 12 months, compared to patients with longer telomeres who showed 89% survival at 60 months. This telomere length threshold was independent of other predictive markers used in breast cancer including ER, PGR, HER2 status, NPI, or grade, and was the dominant variable in multivariate analysis demonstrating the prognostic power of telomere length in this cancer type [126].

Other cancers for which short telomere length can be used as a prognostic indicator include non-small-cell lung cancer, myelodysplastic syndromes (MDS) and multiple myeloma [127,128,129,130]. This finding was particularly striking in Multiple Myeloma, considering median telomere length was measured from a mixed population of cells extracted from bone marrow [129]. The contribution of the myeloma CD138^+^ plasma cell subsets with shorter telomere-length profiles, within the mixed population was sufficient to reduce mean telomere length and allow prognostic discrimination [129].

The above examples are consistent with the idea of telomere length loss and subsequent dysfunction as being a precursor to tumour progression. Other studies indicate that the converse may be true, where for some cancers, longer telomere length predicts poor clinical outcome [131,132,133,134,135,136,137]. This has been demonstrated in oesophageal cancer where patients with a relative telomere length (RTL) greater than 1.22, as measured qPCR, have a shorter median survival time than patients with an RTL under this threshold [131].

Poorer clinical prognosis has been seen in patients with longer leukocyte telomere lengths for a number of cancers: in prostate cancer long telomere length was found to be an independent negative factor for both metastasis-free survival and PC-specific death when evaluated together with established risk factors (tumour stage, Gleason score, and serum PSA) [132]. Similar findings were also observed in breast, kidney, melanoma and hepatocellular carcinoma patients where patients with long leukocyte telomere length displayed a poorer outcome [133,134,135,136,137]. These findings on leukocyte telomere length however, do not necessarily conflict with the model of short dysfunctional telomeres in the developing tumour clone, driving tumour progression through the generation of large scale genomic instability and subsequent clonal evolution. Instead an “immunohypothesis” has been put forward, suggesting that longer leukocyte telomere length may arise in patients with a suppressed immune system, leading to fewer cell divisions and reduced telomere shortening [131]. In support of this hypothesis, a significant correlation between leukocyte telomere length and peripheral levels of immunosuppressive regulatory T cells (Tregs) has been found in kidney cancer patients and hepatocellular carcinoma [136,138].

U-shaped associations have also been observed between telomere length and cancer progression. In a recent case-control study in the Chinese population, both short and extremely long telomeres were found to be risk factors in oesophageal squamous cell carcinoma (ESCC) [139]. A similar scenario has also been found in glioma and pancreatic adenocarcinoma [140,141].

There is a clear need to accurately stratify cancer patients by clinical outlook as close to diagnosis as possible, thereby providing important information to patients, their clinicians and funding agencies, particularly with the advent of expensive targeted therapies. Telomere length shows great promise as a prognostic and predictive marker for certain cancers, though the sometimes contrasting results in telomere length association studies suggest a cautious outlook should be adopted. The influence of telomere length on driving oncogenesis may differ across cancer types, each with distinct phenotypes resulting in diverse effects on survival and modulating the prognostic potential of telomere length. Equally, study design parameters, experimental approach and analysis method may also influence the relationship between telomere length and cancer progression [43]. Several recent meta-analyses of associations between telomere length and cancer prognosis conclude that standardisation of methodology and validation are required, as well as larger prospective studies of specific cancer types to better evaluate the role of telomere length both before and after cancer diagnosis [22,142,143].

## 7. Conclusions

Critically shortened telomeres, rendered dysfunctional by replicative erosion or stochastic deletion, play a central role in driving oncogenesis by generating genomic rearrangements that disrupt oncogene or tumour suppressor pathways, and by generating clonal diversity which fosters genome evolution through natural selection. Recent studies using high-throughput sequencing suggest that the complexity of telomere-induced genome instability may be have been greatly underappreciated, with extremely convoluted and intricate genomic configurations now being reported [100,112,144]. The importance of telomere biology to cancer progression has also been emphasised by a growing number of studies that have correlated short telomere lengths with clinical prognosis. Despite these considerable achievements, quantifying telomere length dynamics remains technically challenging, although emerging technologies promise to address these difficulties.
